# Graphene/Chalcogenide Heterojunctions for Enhanced Electric-Field-Sensitive Dielectric Performance: Combining DFT and Experimental Study

**DOI:** 10.3390/nano16020128

**Published:** 2026-01-18

**Authors:** Bo Li, Nanhui Zhang, Yuxing Lei, Mengmeng Zhu, Haitao Yang

**Affiliations:** 1 Electric Power Research Institute of Yunnan Power Grid Co., Ltd., Kunming 650217, China; 2 Yunnan Provincial Key Laboratory of Green Energy and Digital Power Measurement, Control and Protection, Kunming 650217, China; 3 Kunming Power Supply Bureau of Yunnan Power Grid Co., Ltd., Kunming 650217, China; 4 School of Electrical Engineering, Beijing Jiaotong University, Beijing 100044, China

**Keywords:** graphene, two-dimensional chalcogenides, heterojunction, DFT, electric-field sensing

## Abstract

Electric-field-sensitive dielectrics play a crucial role in electric field induction sensing and related capacitive conversion, with interfacial polarization and charge accumulation largely determining the signal output. This paper introduces graphene/transition metal dichalcogenide (TMD) (MoSe_2_, MoS_2_, and WS_2_) heterojunctions as functional fillers to enhance the dielectric response and electric-field-induced voltage output of flexible polydimethylsiloxane (PDMS) composites. Density functional theory (DFT) calculations were used to evaluate the stability of the heterojunctions and interfacial electronic modulation, including binding behavior, charge redistribution, and Fermi level-referenced band structure/total density of states (TDOS) characteristics. The calculations show that the graphene/TMD interface is primarily controlled by van der Waals forces, exhibiting negative binding energy and significant interfacial charge rearrangement. Based on these theoretical results, graphene/TMD heterojunction powders were synthesized and incorporated into polydimethylsiloxane (PDMS). Structural characterization confirmed the presence of face-to-face interfacial contacts and consistent elemental co-localization within the heterojunction filler. Dielectric spectroscopy analysis revealed an overall improvement in the dielectric constant of the composite materials while maintaining a stable loss trend within the studied frequency range. More importantly, calibrated electric field induction tests (based on pure PDMS) showed a significant enhancement in the voltage response of all heterojunction composite materials, with the WS_2_-G/PDMS system exhibiting the best performance, exhibiting an electric-field-induced voltage amplitude 7.607% higher than that of pure PDMS. This work establishes a microscopic-to-macroscopic correlation between interfacial electronic modulation and electric-field-sensitive dielectric properties, providing a feasible interface engineering strategy for high-performance flexible dielectric sensing materials.

## 1. Introduction

In high-voltage engineering and power electronics environments, accurate measurement and mapping of electric field distribution are crucial because electric field distribution is closely related to insulation design margin, surface charge accumulation, partial discharge risk, and transient overvoltage characteristics in actual substations and equipment. Compact capacitive electric field sensors and their related measurement strategies have been widely studied in smart grid applications and high-voltage monitoring due to their wide bandwidth advantage and non-contact deployment [1,2,3]. The demand for portable sensing in high-voltage operating environments has also driven the development of flexible, lightweight, and highly reliable sensing materials that can be integrated into conformal packages or distributed sensor nodes [4,5]. Among candidate material platforms for flexible sensing, polymer dielectric materials (such as silicone rubber and polydimethylsiloxane, PDMS) have attracted much attention due to their mechanical compliance, ease of processing, chemical stability, and compatibility with large-area manufacturing. Pure elastomeric dielectric materials typically have relatively low dielectric constants, which limit the polarization intensity and thus the voltage response amplitude (or capacitance change) under a given external electric field excitation [6,7]. How to enhance dielectric polarization and modulate the dielectric response of polymer matrices without sacrificing mechanical flexibility and low-loss characteristics has been a long-term research topic in the fields of electrical insulation and flexible electronics [8]. Currently, a widely adopted strategy is to introduce functional fillers into polymer matrices to form dielectric composites. Traditional ceramic fillers can improve the dielectric constant but usually require high addition amounts, which may reduce the material’s flexibility, processing performance, and interfacial reliability [9]. In contrast, conductive or semiconductor nanofillers with high aspect ratios can generate significant interfacial polarization and micro-capacitor network effects at relatively low addition amounts, thereby achieving significant dielectric constant enhancement under alternating electric fields. This behavior is usually related to Maxwell-Wagner-Silas (MWS) interfacial polarization, where charge carriers accumulate at heterogeneous interfaces due to conductivity/dielectric constant mismatch, resulting in additional polarization contributions beyond the intrinsic response of the polymer [10,11,12].

Transition metal dichalcogenides (TMDs, such as MoS_2_, MoSe_2_, and WS_2_) are important building blocks for constructing such heterostructures. Unlike graphene, monolayer or few-layer transition metal dichalcogenides (TMDs) typically exhibit semiconductor properties, and their band structure varies with thickness, thus attracting extensive research in electronics and sensing [13]. Fundamental studies have shown that atomically thin molybdenum disulfide (MoS_2_) transforms into a direct bandgap semiconductor in the monolayer limit, which has sparked widespread interest in the electronic structure of two-dimensional TMDs and their engineering application prospects [14]. Further research has highlighted the chemical diversity and tunable electronic properties of layered TMD nanosheets, thus supporting various interface control functions. When graphene is combined with semiconductor TMDs, the resulting graphene/TMD van der Waals heterostructure can exhibit significant interfacial charge transfer, band alignment effects, and contact type transitions (Schottky/Ohmic properties), which are crucial for carrier redistribution and polarization under external fields [15,16]. Both experimental and theoretical studies have reported the band arrangement and microband gap characteristics in MoS_2_/graphene van der Waals heterostructures, indicating that electronic coupling and interface-induced charge transfer can be directly reflected in the band structure and density of states [17]. Furthermore, the contact and charge transfer effects in graphene–MoS_2_ heterostructures significantly influence transport behavior, suggesting that the interface electronic structure is an effective means of regulating macroscopic responses [18,19]. These results indicate that graphene/TMD heterojunctions can serve as functional dielectric fillers, enhancing polarization response by controlling interface electronic interactions, rather than solely relying on random percolation networks.

Based on these considerations, this work focuses on graphene/chalcogenide (MoS_2_, MoSe_2_, WS_2_) heterojunctions, aiming to establish a clearer micro-macroscopic relationship between the interface electronic structure and field-sensitive dielectric properties in polymer composites. This paper employs first-principles calculations to quantify the interfacial bonding characteristics, charge density redistribution, and band structure modulation in a graphene/transition metal dichalcogenide (TMD) heterojunction model, providing a microscopic basis for explaining the polarization enhancement mechanism. Corresponding heterojunction composite fillers were synthesized and embedded in a polydimethylsiloxane (PDMS) matrix to prepare dielectric composites, followed by structural characterization and calibration of the electric field response. By combining the calculated results with experimentally measured dielectric and electric-field-induced voltage responses, this study aims to elucidate how van der Waals heterojunctions amplify the effective dielectric response under an external electric field and provide guidance for the design of high-performance electric-field-sensitive dielectric composites and sensing materials.

## 2. Experimental and DFT Calculation Methods

### 2.1. Materials Synthesis

**Figure 1:**
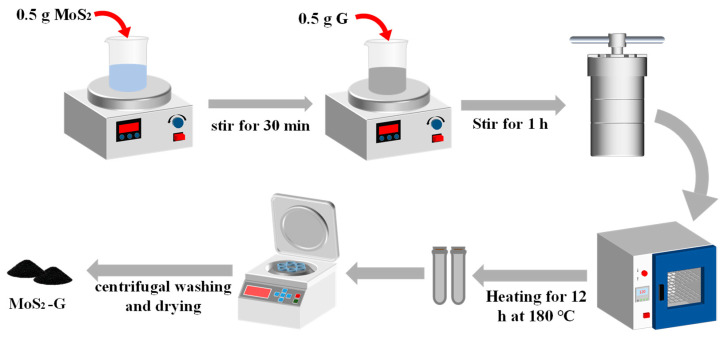
Preparation process of graphene and MoS_2_ composite materials.

Graphene nanosheets and transition metal dichalcogenide (TMD) nanoparticles (MoS_2_, MoSe_2_, and WS_2_), along with all other reagents, were purchased from Aladdin Reagents (Shanghai, China) Co., Ltd. The graphene nanosheets had a lateral diameter of 5–10 μm and a thickness of 5 nm. The TMD particles had an average grain size of 100–500 nm. Graphene and the three TMD materials were synthesized using a hydrothermal method with identical steps. A detailed description is provided using the MoS_2_-G heterojunction as an example: First, 0.5 g of MoS_2_ powder was dispersed in 30 mL of deionized water and magnetically stirred for 30 min at room temperature to form a homogeneous suspension. Then, 0.5 g of graphene was slowly added to the suspension, and stirring continued for 1 h to ensure homogeneity. The mixture was then transferred to a 200 mL polytetrafluoroethylene-lined high-pressure reactor. The sealed autoclave was placed in a constant temperature oven at 200 °C for 12 h. After the heating reaction was completed, the reactor was removed and allowed to cool naturally to room temperature. The collected precipitate was washed three times each with deionized water and anhydrous ethanol and then centrifuged at 8000 rpm. Finally, the MoS_2_-G powder was dried under vacuum at 60 °C for 12 h to obtain MoS_2_-G heterojunction powder. The entire synthesis process is shown in Figure 1.

**Figure 2:**
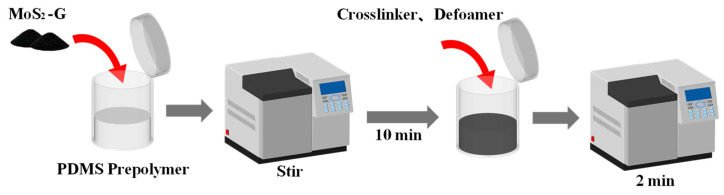
Experimental Process Diagram of Heterojunction Material/PDMS Prepolymer Blending and Curing.

The composite material formed by heterojunction powder and PDMS prepolymer was also prepared by solution blending. PDMS matrix and curing agent (Sylgard 184 A) were mixed at a mass ratio of 10:1, and then the synthesized graphene/TMD heterojunction powders (MoSe_2_-G, MoS_2_-G, and WS_2_-G) were added to the PDMS mixture. To ensure a fair comparison of the inherent properties of different heterostructures, the filler content of all composite samples was fixed at 5.0 wt%. The mixture was mechanically stirred for 30 min, followed by ultrasonic treatment for 1 h to ensure uniform filler dispersion. Finally, the mixture was degassed in a vacuum chamber to remove residual bubbles and cured in an oven at 80 °C for 2 h to obtain a flexible composite film, as shown in Figure 2.

### 2.2. Material Characterization

To verify the quality of graphene heterojunction materials as functional fillers and the effectiveness of the composite structure and to thoroughly investigate their microstructure, interface bonding state, and elemental distribution characteristics, this study employed a Zeiss Gemini SEM 300 field emission scanning electron microscope (FE-SEM) along with the Oxford Instruments Xplore 30 energy-dispersive X-ray spectroscopy (EDS) for systematic characterization. The sample preparation procedure for characterization is as follows: The homemade heterojunction powder is evenly dispersed in anhydrous ethanol and sonicated for 15 min. An appropriate amount of the dispersion is then dropped onto the sample stage and allowed to dry at room temperature. Subsequently, using a Leica-EM ACE600 ion sputtering instrument, a layer of Au/Pd alloy film approximately 5 nm thick is coated on the sample surface, completing the preparation for characterization.

### 2.3. Dielectric Spectroscopy and Electric-Field Sensing Tests

Dielectric spectrum testing was conducted using the centralized circuit method. To avoid capacitance interference errors caused by air gaps, flexible copper foil contact electrodes were used during the testing process. The flexible copper foil contact electrodes were made from double-sided conductive copper foil with a diameter of 30 mm. The core testing equipment uses the Tonghui TH2830 LCR digital bridge, paired with a dedicated tweezer-type test fixture for measurements. The experiment also includes auxiliary equipment: a high-precision microscopic thickness gauge is used for accurate thickness measurement of the samples; a vacuum drying oven is used for pre-treatment of the samples before testing. Through the coordinated use of the core and auxiliary equipment, a complete dielectric spectroscopy testing system has been established. The preparation process of dielectric spectroscopy test samples is as follows: First, a PDMS184-specific release agent is sprayed onto a glass substrate. Using a wire rod coater, a uniform film with a thickness of 100 ± 10 μm is produced. After curing in a vacuum at 80 °C for 2 h, the film is placed in an 80 °C water bath and gently peeled off with the help of the release agent. The film is then cut into 40 mm × 40 mm square pieces using a cutting tool. To form a standard parallel plate capacitor structure, attach 30 mm circular double-sided conductive copper tape at the geometric center of both sides of the sample as electrodes, ensuring full contact between the electrodes and the sample surface without any trapped air bubbles.

To verify the modification effects of the three chalcogen compounds, according to the material preparation process described above, five graphene heterojunction composite samples were prepared for testing: PDMS, G/PDMS, MoS_2_-G/PDMS, MoSe_2_-G/PDMS, and WS_2_-G/PDMS, with G/PDMS serving as the control group to assess the modification effects of the heterojunctions. In terms of test parameter settings, the frequency scan range is set from 50 Hz to 50 kHz, using a logarithmic sweep mode, and five characteristic frequency points are measured for every tenfold frequency. The test signal uses an AC voltage of 1.0 Vrms. Before testing the samples, a high-precision microscopic thickness gauge is used to measure the thickness at three points, and an average value is taken. The samples are then placed in a tweezer-type test fixture to start automatic frequency sweep testing, and the measurement data is recorded. Each sample is tested three times to ensure the consistency of the results. During the data processing, the capacitance value and the dissipation factor are directly read from the LCR digital bridge, while the phase angle at each frequency point is also recorded. The tested dielectric constant can be expressed as: The dielectric constant (ε) is calculated using Equation (1), where C is the capacitance loss factor, and both the loss factor (D) and the phase angle at the loss frequency can be directly obtained using an LCR digital bridge. d is the sample thickness, $\varepsilon_{0}$ represents the vacuum dielectric constant (8.85 × 10^−12^ F/m), and A is the electrode area.

(1) $$\varepsilon =\frac{Cd}{\varepsilon_{0}A}$$

**Figure 3:**
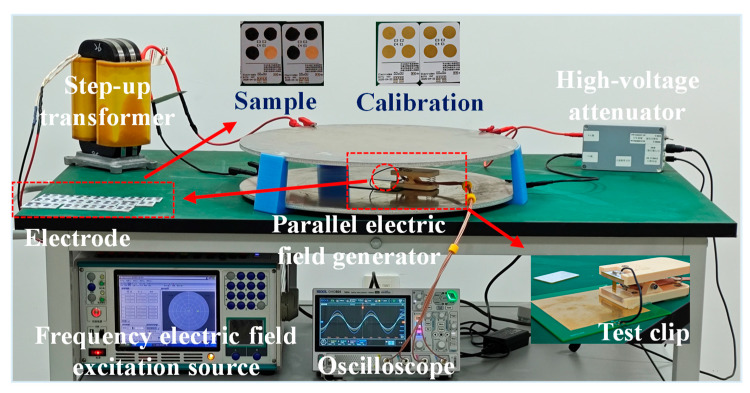
Experimental process diagram of electric field sensing performance test.

The dielectric loss factor tan δ can be directly read from the measured value. The electric field induction performance testing system and testing scheme are shown in Figure 3.

To evaluate the electric field sensing response characteristics of materials, a parallel plate electrode electric field generation and testing system was set up. It can produce a wide range of uniform and controllable standard electric fields, measuring the voltage output of dipole electric-field-induced sensors in functional thin film materials, providing an experimental basis for their application in power line electric field measurements. The system consists of a parallel plate electric field generation device, a sensing unit, and a signal acquisition unit. The generation device uses 500 mm diameter parallel circular aluminum electrodes, with the upper plate connected to an adjustable high-voltage AC source composed of a programmable AC power supply and a step-up transformer, and the lower plate grounded through a protective resistor; the sensing unit is a custom triple-electrode piece with calibrated electrodes, supporting quick installation and removal as well as simultaneous testing of multiple samples. The sample was tested under stress-free conditions (zero external mechanical pressure); a multi-channel digital oscilloscope is responsible for signal acquisition, with one channel monitoring the excitation electric field through a high-voltage differential probe, and the other three channels using high-impedance single-ended probes to collect the sensing unit’s response signals, which are then uploaded to a computer for processing. The testing procedure is as follows: Maintaining a 50 Hz test frequency, starting the programmable AC power supply, generating a preset high-voltage AC signal through a step-up transformer, applying an electric field strength scanned from 0 to 5 kV/cm to the upper electrode, while simultaneously grounding the lower electrode to form a uniform alternating electric field. The generated electric field can be calculated using the following formula:

(2) $$E=\frac{V}{d1}$$

V is the voltage between the electrodes (monitored using a high-voltage differential probe), and d1 is the distance between the electrodes (a fixed value). Before testing, perform a blank calibration to record the baseline readings of the probe without any samples, in order to eliminate the effects of parasitic capacitance and electromagnetic noise; precisely align and place a graphene/PDMS composite film with a thickness of 100 ± 10 μm in the sensitive area of the electrode, fix the position with a mechanical fixture, remove bubbles under vacuum adsorption, and cure for 2 h in a vacuum environment at 80 °C; during the formal test, synchronously collect the induced charge signals, repeating each experiment 5 times and taking the average.

### 2.4. DFT Calculations Details

Geometry optimization and energy calculations were performed based on first-principles theory, using the Dmol3 module integrated in the Materials Studio (MS) software package (2019) [20]. The electron exchange–correlation interaction was described by the Perdew–Burke–Ernzerhof (PBE) functional within the framework of generalized gradient approximation (GGA) [21,22]. For the treatment of core electrons and electron pseudopotentials, DFT semi-core pseudopotentials (DSPP) and the dual numerical basis set with polarization functions (DNP) were adopted [23,24,25]. Dispersion corrections, encompassing van der Waals forces and long-range interactions, were incorporated via the DFT method [26,27]. The k-point sampling density in the Brillouin zone was set to 7 × 7 × 1 for both geometry optimization and electronic structure calculations. The convergence criteria for optimization were specified as follows: energy tolerance of 10^−5^ Ha, maximum force of 2 × 10^−3^ Ha/Å, and maximum displacement of 5 × 10^−3^ Å [28]. For static electronic structure calculations, the global orbital cutoff radius, self-consistent field (SCF) energy tolerance, and smearing parameter were set to 5 Å, 10^−6^ Ha, and 0.005 Ha, respectively [29,30].

In order to build an effective heterojunction model, a 4 × 4 × 1 graphene supercell was first established, in which the lattice constant of graphene is a = b = 9.84 Å. In order to successfully build a heterojunction structure between three two-dimensional chalcogen compounds (MoS_2_, MoSe_2_, WS_2_) and graphene, it is necessary to ensure that the lattice mismatch rate is less than 5%. The calculation formula of the lattice mismatch rate is defined in Equation (3), where $a_{1}$ and $a_{2}$ are the lattice constants of the two models, respectively. 3 × 3 × 1 MoS_2_, 3 × 3 × 1 MoSe_2_ and 3 × 3 × 1 WS_2_ supercells were established, with lattice constants of 9.576 Å (MoS_2_), 9.966 Å (MoSe_2_) and 9.552 Å (WS_2_), respectively. After calculation, it was found that the three established two-dimensional chalcogenide and graphene structures can meet the requirement of 5% lattice mismatch rate. To improve computational efficiency and capture the inherent charge transfer behavior of the interface, we model the heterostructure using monolayer graphene and monolayer transition metal dichalcogenides. For the established periodic heterojunction structure, a 20 Å vacuum layer was added along the c-axis to avoid the influence of layer-to-layer interactions on the calculation results.

(3) $$△=\frac{2\left|a_{1}-a_{2}\right|}{a_{1}+a_{2}}$$

To achieve a quantitative characterization of the interaction strength between graphene and three materials, the concept of binding energy is introduced, defined as shown in the formula [30].

(4) $$E_{b}=E_{H}-E_{O}-E_{G}$$

*E_b_* is the binding energy, *E_H_* is the total energy of the heterojunction formed by graphene and the target material, *E_O_* is the individual energy of the components in the heterojunction excluding graphene, and *E_G_* is the energy of graphene in the heterojunction. 

When graphene is assembled with the three TMD monolayers to form heterojunctions, interfacial electron redistribution inevitably takes place. The amount of charge transfer ($Q_{t}$) was quantified using Mulliken population analysis and calculated according to Equation (5). In this definition, $Q_{a}$ denotes the net Mulliken charge of a selected component (graphene or the TMD layer) within the relaxed heterostructure, which was obtained by summing the Mulliken atomic charges of all atoms belonging to that component. $Q_{b}$ represents the net Mulliken charge of the same component in the corresponding isolated monolayer, computed using the same supercell and identical calculation parameters. The sign of $Q_{t}$ indicates the transfer direction: $Q_{t}<0$ implies electrons flow from graphene to the TMD layer, whereas $Q_{t}>0$ suggests electron donation from the TMD layer to graphene [30].

(5) $$Q_{t}=Q_{a}-Q_{b}$$

## 3. Results and Discussions 

### 3.1. Characterization of Material Properties

**Figure 4:**
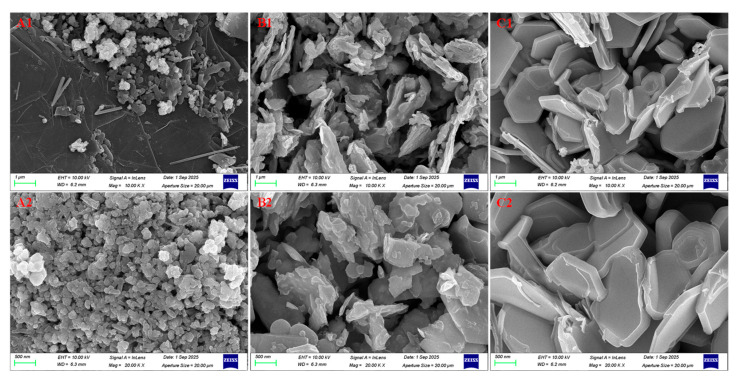
SEM images of graphene/TMD heterojunction powders at two magnifications. (**A1**,**A2**) MoSe_2_-G, (**B1**,**B2**) MoS_2_-G, (**C1**,**C2**) WS_2_-G. The top row (**A1**–**C1**) shows low-magnification images (scale bar: 1 µm), and the bottom row (**A2**–**C2**) shows high-magnification images (scale bar: 500 nm).

Figure 4 presents the SEM images of the three graphene/TMD heterojunction powders at varying magnifications. Overall, all samples exhibit typical hybrid morphologies composed of sheet-like graphene and chalcogenide domains, confirming the successful construction of heterostructure powders with abundant interfacial contact. As illustrated in Figure 4(A1,A2), the MoSe_2_-G heterojunction features a graphene-like layered substrate decorated with numerous MoSe_2_ aggregates. In the lower-magnification view (A1), these aggregates appear as densely populated granular clusters uniformly dispersed on the sheet surface. The higher-magnification image (A2) further reveals that these domains are primarily submicron-sized clusters composed of smaller nanoscale building blocks, with lateral dimensions typically ranging from 200 to 500 nm. Such a morphology implies a high density of heterointerfaces, which is favorable for enhancing interfacial polarization.

In contrast, the microstructure of the MoS_2_-G composite (Figure 4(B1,B2)) is dominated by irregular flake-like fragments and stacked lamellar domains. These flakes exhibit wrinkled edges, appearing closely packed and interconnected to form a porous but compact structure. At higher magnification, the lamellar nature becomes evident, with MoS_2_ lamellae generally falling within the size range of 500 nm to 1 μm. This stacked layered morphology suggests that the MoS_2_ is integrated with graphene through intimate contact rather than existing as isolated particles, facilitating charge accumulation at the interfaces. Distinctively, the WS_2_-G sample (Figure 4(C1,C2)) displays well-defined platelet-like microflakes with clear edges. The low-magnification image reveals abundant platelets with micrometer-scale dimensions, many of which exhibit a quasi-hexagonal morphology, characteristic of high-quality layered WS_2_ crystals. As shown in the high-resolution view (C2), these platelets possess sharp step edges and lateral sizes reaching approximately 1–2 μm. The coexistence of these large WS_2_ nanoplates and sheet-like graphene indicates the formation of extensive face-to-face contact regions, providing extended heterointerfaces crucial for the dielectric response. In summary, the SEM observations reveal a clear morphological evolution: MoSe_2_-G forms granular clusters, MoS_2_-G exhibits stacked lamellae, and WS_2_-G presents large hexagonal platelets. Despite these variations, all structures demonstrate intimate coupling between the graphene network and the TMD components.

**Figure 5:**
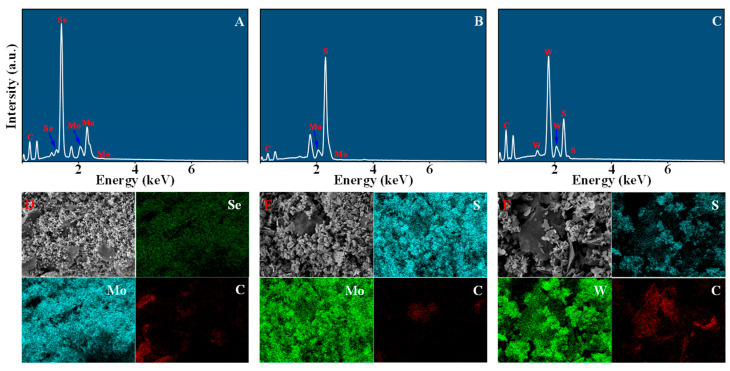
EDS characterization results of graphene/TMD heterojunction powders. (**A**–**C**) are the EDS spectra of MoSe_2_-G, MoS_2_-G, and WS_2_-G, respectively. (**D**–**F**) are the corresponding SEM images and elemental distribution maps, showing the spatial distribution of Se/Mo/C, S/Mo/C, and S/W/C in MoSe_2_-G (**D**), MoS_2_-G (**E**), and WS_2_-G (**F**), respectively.

Figure 5 shows the comprehensive EDS characterization results of the synthesized graphene/TMD heterojunction powder, including EDS spectra (A–C) and corresponding elemental mapping images (D–F). Overall, the results confirm the coexistence of graphene carbon signals and characteristic chalcogenide elements, providing direct chemical evidence for the successful construction of high-purity heterojunction structures. For the MoSe_2_-G sample, the EDS spectrum in Figure 5A shows distinct peaks associated with Mo and Se, as well as carbon (C) peaks from the graphene framework. The corresponding elemental mapping images in Figure 5D show strong spatial correlation, with Mo and Se signals located in the same region, confirming the integrity of the MoSe_2_ phase. Furthermore, the broad distribution of the C signal indicates that the MoSe_2_ aggregates are firmly anchored on the graphene substrate, suggesting the formation of a stable heterojunction interface at the microscale.

Similarly, for the MoS_2_-G sample (Figure 5B), distinct S and Mo peaks were observed, accompanied by a C signal. The elemental distribution map in Figure 5E further demonstrates that the Mo and S components are tightly bound to the graphene network, rather than existing as isolated particles detached from the carbon framework. This overlap directly confirms that the MoS_2_ domains have been successfully integrated into the heterostructure, thus ensuring effective electrical contact. For the WS_2_-G sample, the spectrum in Figure 5C shows characteristic W and S peaks. The elemental distribution map in Figure 5F confirms the uniform co-distribution of W and S elements within the scanned region, which perfectly matches the widely distributed carbon elemental distribution map, confirming the successful synthesis of WS_2_–graphene heterojunction powder without detected impurities. In summary, the EDS spectra and elemental distribution maps consistently confirm the chemical composition and purity of each composite material. Crucially, the observed elemental colocalization indicates a tight microstructural integration between the two-dimensional graphene sheets and the transition metal dichalcogenide (TMD) regions. This unique structural feature is conducive to maximizing the effective heterogeneous interface area, which is expected to significantly enhance interfacial polarization and improve the dielectric response of subsequent polydimethylsiloxane (PDMS) composites.

### 3.2. Dielectric and Electric-Field Performance Test Results

**Figure 6:**
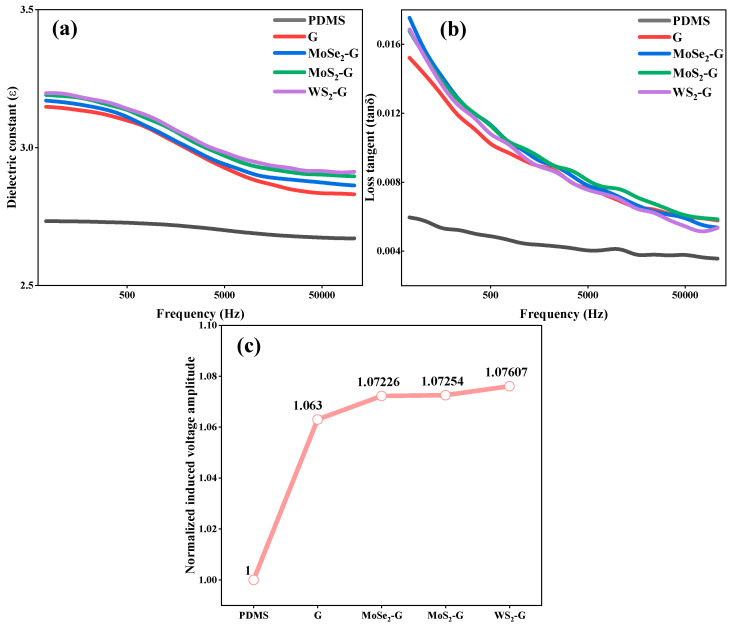
Frequency-dependent dielectric and electric-field response characteristics of pure PDMS and graphene-/heterojunction-filled PDMS composites. (**a**) Real part of the dielectric constant as a function of frequency. (**b**) Dielectric loss tangent as a function of frequency. (**c**) Normalized electric-field-induced voltage amplitude of different samples, with pure PDMS used as the reference (PDMS = 1). Samples include PDMS, graphene/PDMS (G), and graphene/TMD heterojunction composites (MoSe_2_-G, MoS_2_-G, and WS_2_-G).

Figure 6 summarizes the dielectric spectral properties and electric-field-induced voltage output of pure PDMS and PDMS composites filled with graphene and graphene/TMD heterojunctions. Figure 6a shows that the real part of the dielectric constant (ε) of all samples monotonically decreases with increasing frequency, which is typical dielectric dispersion behavior of polymer-based composites. At low frequencies, interfacial polarization and dipole orientation respond more effectively to external alternating electric fields, resulting in relatively high ε. As the frequency increases, these polarization processes gradually become less responsive, leading to a gradual decrease in ε. Compared to pure PDMS, the introduction of graphene and graphene/TMD heterojunction fillers improves ε across the entire frequency range, indicating that the conductive/semiconductor two-dimensional filler and its large-area interface provide additional polarization sites and promote charge accumulation at the interface. Figure 6b shows the corresponding dielectric loss tangent (tan δ) as a function of frequency. Similar to ε, tan δ also decreases with increasing frequency, indicating that conduction loss and interfacial polarization loss dominate at low frequencies while weakening at high frequencies. Notably, although the tan δ value of the composite material is higher than that of pure PDMS (consistent with the introduction of more charge carriers and interfacial relaxation processes), the overall loss remains relatively low without any abnormal peaks, indicating that the filler has been adequately incorporated without causing severe leakage paths or unstable dielectric relaxation. Furthermore, the tan δ curves of different heterojunction composites are close to each other, which is reasonable given their identical matrix (PDMS) and similar filler content; therefore, the main differences stem from subtle variations in interfacial electronic coupling rather than drastic changes in bulk conductivity.

To directly evaluate the electric field sensing capability, Figure 6c compares the electric-field-induced voltage amplitudes of different samples using pure PDMS (PDMS = 1.00) as a normalized reference. This normalization method eliminates the influence of inter-device differences and highlights the relative enhancement effect brought by functional fillers. The results show that the introduction of graphene has a significant enhancement effect, with a normalized voltage amplitude of approximately 1.063, confirming that the introduction of a two-dimensional conductive network and interfacial polarization can improve the macroscopic electric field response. More importantly, all three graphene/TMD heterojunction fillers further increased the normalized voltage amplitude to a higher level (approximately 1.07–1.08), indicating that the construction of the graphene–chalcogenide interface provides additional interfacial polarization centers besides graphene itself. Among them, the WS_2_-G/PDMS composite material exhibits the highest response (=1.07607), which is 7.607% higher than that of pure PDMS. The overall ranking in Figure 6c shows that heterojunction engineering can significantly and stably improve electric field sensitivity. Combining the dielectric spectrum trend (Figure 6a,b) and voltage response comparison (Figure 6c), the enhancement mechanism can be attributed to the increased density of interfacial polarization sites and the enhanced charge accumulation ability brought about by the graphene/TMD heterojunction structure.

### 3.3. Analysis of Calculation Results

**Figure 7:**
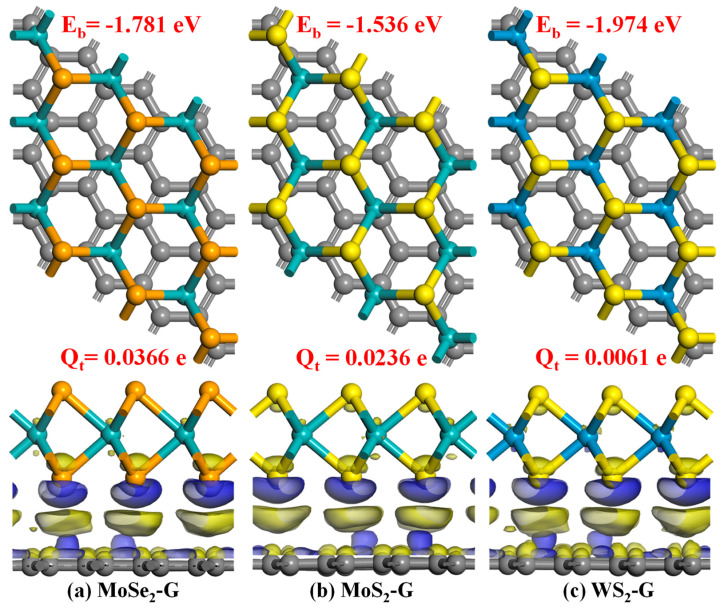
Optimized atomic structures and interfacial charge redistribution of graphene/TMD heterojunctions obtained from DFT calculations. The **upper** panels show the top-view geometries of three representative graphene–chalcogenide heterostructures (left to right), with the corresponding binding energies labeled. The **lower** panels present the side-view structures together with the charge-density difference surfaces at the interface, where charge accumulation and depletion regions are indicated by different colors. The net interfacial charge transfer for each heterojunction is also given.

Figure 7 illustrates the optimized structural forms and interfacial electron redistribution behavior of three graphene/TMD heterojunctions (MoSe_2_-G, MoS_2_-G, and WS_2_-G). The optimized structures show that the graphene and chalcogenide layers maintain a typical face-to-face stacking arrangement. No chemical bonds were observed in any of the three systems, indicating that the two different interfaces are primarily formed through van der Waals interactions. The calculated binding energies further confirm the thermodynamic feasibility of heterojunction formation. All three systems exhibit negative binding energy values, indicating that graphene/TMD stacking is energy-favorable and can spontaneously form without external energy input. Although the binding energy values are generally comparable, significant differences in interfacial coupling strength are observed. The WS_2_–graphene heterojunction exhibits the strongest binding energy and the most negative cohesive energy value (−1.974 eV), indicating stable interfacial adhesion in the studied structures. The other two heterojunctions exhibit slightly smaller binding energy values (−1.781 eV (MoSe_2_-G) and −1.536 eV (MoS_2_-G)), indicating that the interfacial registration details and inherent electronic properties of different chalcogenides affect the overall stability of the stacked interface.

Besides structural stability, Figure 7 also reveals a significant redistribution of interfacial charge during heterojunction formation. The lower figure shows the charge density difference iso-surface, where two distinct regions (yellow and blue) appear in the graphene–TMD contact region, representing electron accumulation and electron depletion, respectively. This suggests that stacking leads to a redistribution of electrons near the interface, rather than a uniform charge change between layers. Notably, the charge rearrangement is concentrated in the region near the interface (near the interface between the graphene upper surface and the chalcogenide layer docking surface), indicating the formation of an interfacial polarized charge distribution. Quantified net charge transfer values provide a comparison of the redistribution of electron intensity at the interface in the three systems. The MoSe_2_–graphene heterojunction exhibits the most significant charge transfer magnitude (Q_t_ = 0.0366 e), indicating its strongest interfacial electron rearrangement. MoS_2_–graphene heterojunctions exhibit moderate charge transfer amplitudes, with a charge transfer value of 0.0236 e. In contrast, although the WS_2_–graphene system possesses the strongest binding energy, its net charge transfer is relatively small (Q_t_ = 0.0061 e). In summary, the structural and electronic evidence shown in Figure 7 together suggest that the graphene/TMD heterojunctions are formed through stable van der Waals stacking, resulting in interfacial charge polarization. The differences in binding energy, interfacial charge redistribution intensity, and net charge transfer values among MoSe_2_-G, MoS_2_-G, and WS_2_-G indicate different interfacial coupling properties, providing a microscopic basis for understanding the experimentally observed variations in dielectric behavior and electric field response in the corresponding graphene/TMD-PDMS composites.

**Figure 8:**
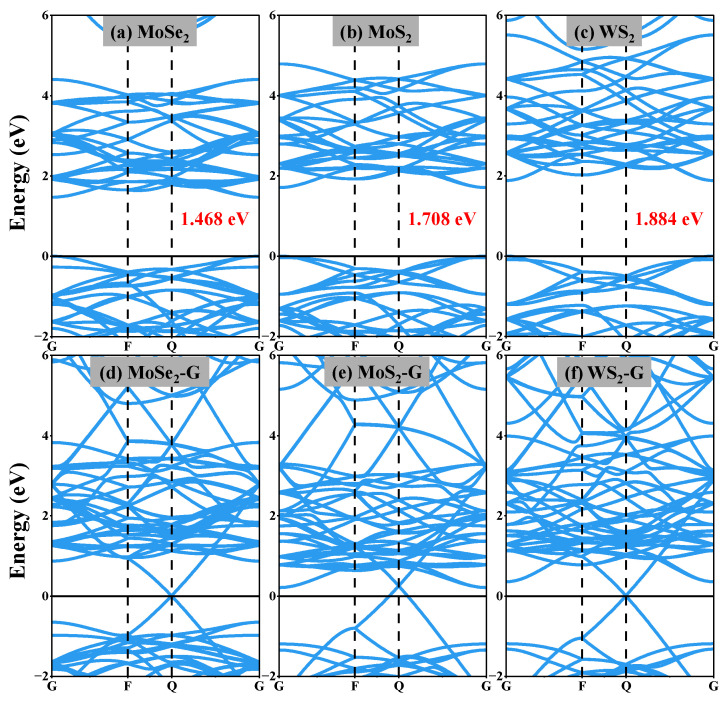
Band structures obtained from DFT calculations. (**a**–**c**): Intrinsic band structures of monolayer MoSe_2_, MoS_2_, and WS_2_, respectively, the valence-band maximum (VBM) is set to 0 eV to indicate the intrinsic band gaps. (**d**–**f**): Band structures of the corresponding graphene/TMD heterojunctions (MoSe_2_-G, MoS_2_-G, and WS_2_-G), where the Fermi level is set to 0 eV.

Figure 8 shows the band structures of the original monolayer transition metal dichalcogenides (TMDs) (MoSe_2_, MoS_2_, and WS_2_) and their corresponding graphene-based heterojunctions obtained by DFT calculations. Figure 8a–c show the intrinsic electronic band structures of monolayer MoSe_2_, MoS_2_, and WS_2_, respectively. The calculated band gap values are 1.468 eV for MoSe_2_, 1.708 eV for MoS_2_, and 1.884 eV for WS_2_, indicating that the band gap size order is MoSe_2_ < MoS_2_ < WS_2_. The band dispersion near the valence band top and conduction band bottom also reflects the differences in the intrinsic electronic structure among these chalcogenides. Figure 8d–f illustrates the band structures of the corresponding graphene/TMD heterojunctions (MoSe_2_-G, MoS_2_-G, and WS_2_-G). Compared to the pristine TMD, the heterojunctions exhibit significant band reconstruction and higher band dispersion density. The introduction of graphene causes the bands to cross or approach the Fermi level, implying a more conductive electronic environment than isolated semiconductor TMD layers. A direct comparison of the band diagrams of the pristine TMD and the heterojunctions reveals that constructing the graphene/TMD interface significantly alters the electronic structure. Compared to the intrinsic band gap of monolayer transition metal dichalcogenides (TMDs), the band gap of the heterojunction system is significantly reduced; in some cases, the band edges are even closer to the Fermi level. This modulation indicates that interfacial electronic coupling and charge redistribution at the graphene–TMD interface alter the energy level alignment and the density of electronic states near the electric field. This interface effect is expected to promote charge accumulation and polarization under an applied electric field, thus providing a microscopic explanation for the enhanced dielectric response and improved electric field sensitivity of the graphene/TMD-PDMS composite material observed in experiments.

**Figure 9:**
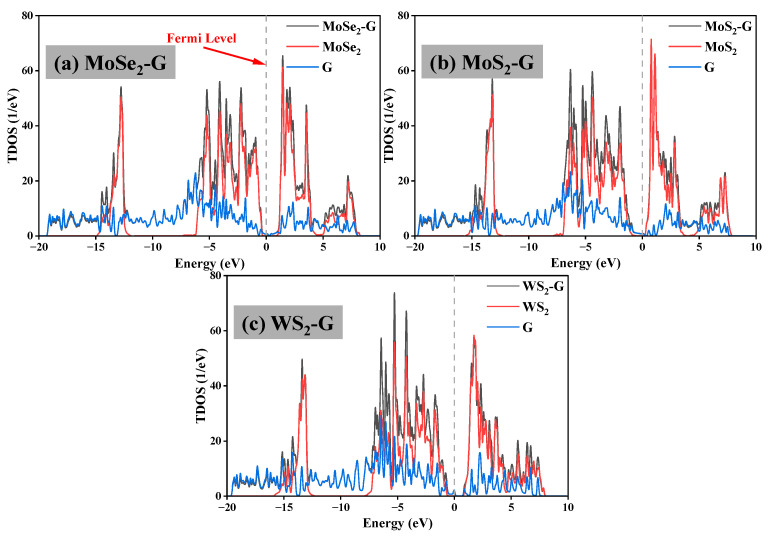
Total density of states (TDOS) of the three graphene/TMD heterojunction systems and their individual components: (**a**) MoSe_2_-G, (**b**) MoS_2_-G, and (**c**) WS_2_-G. In each panel, the TDOS of the heterojunction (black) is compared with those of the corresponding pristine TMD layer (red) and graphene (blue). The Fermi level is set to 0 eV and is indicated by the vertical dashed line.

Figure 9 compares the total density of states (TDOS) of the three graphene/TMD heterojunction systems with their individual components. For the isolated TMD monolayer, the TDOS near the Fermi level is relatively low, consistent with the semiconductor properties observed in the band structure results. In contrast, graphene exhibits a limited number of electronic states near the Fermi level, reflecting its half-metallic properties and the presence of graphene-derived states near the Fermi level. After heterojunction formation, the TDOS of the graphene/TMD system is not a simple linear superposition of the two isolated components. Instead, significant changes occur in the energy region and valence/conduction band regions near the Fermi level, indicating that interfacial electronic coupling and charge redistribution lead to a reconstruction of the overall electronic density of states. In all three heterojunction systems, the TDOS near the Fermi level remains limited, implying that graphene-derived states significantly contribute to the electronic structure near the Fermi level. Compared to pure graphene and TMD, the TDOS of the heterojunction exhibits enhanced and redistributed peak characteristics across multiple energy windows, particularly in the valence band region below 0 eV and the conduction band region above 0 eV. These variations suggest that interfacial interactions induce energy level alignment and hybridization between the graphene and TMD layers, introducing additional electronic states and shifting the TDOS distribution toward the Fermi level. The differences in peak intensity and distribution around the Fermi level across the three systems further indicate that the degree of electronic interaction depends on the type of TMD. These differences are consistent with the variations in interfacial bonding and charge transfer behavior discussed in the heterojunction structural analysis. From a structure-property perspective, the presence and modulation of electronic states near the Fermi level can promote charge accumulation and interfacial polarization under an applied electric field, thus providing a microscopic explanation for the experimentally observed enhancement in the dielectric response and electric-field-induced voltage output of the graphene/TMD-PDMS composite.

## 4. Conclusions

This study successfully prepared and thoroughly analyzed heterojunction composites of graphene with three two-dimensional chalcogenides (MoS_2_, MoSe_2_, WS_2_) through systematic experiments and theoretical calculations. Microstructural characterization confirmed that all three types of heterojunctions formed face-to-face, closely packed interfaces dominated by van der Waals forces. The MoSe_2_-G and MoS_2_-G interfaces exhibited slight elemental interdiffusion and a potential tendency for covalent bonding, whereas the WS_2_-G interface displayed typical van der Waals heterojunction characteristics, with a clear and intact interface. At the macroscopic performance level, all heterogeneous fillers significantly enhanced the dielectric constant of PDMS-based composites and introduced beneficial interfacial polarization effects. In the electric field induction test, the WS_2_-G/PDMS composite material exhibited the best performance, with its normalized induced voltage amplitude increasing by 7.607% compared to pure PDMS, highlighting its great application potential in the field of electric field sensing. Theoretical calculations further reveal the root cause of performance differences at the mechanistic level: density functional theory (DFT) results indicate that the formation of heterojunctions is a spontaneous process, with all interaction energies being negative, among which the WS_2_-G system has the strongest binding (−1.974 eV). Calculations confirm the presence of charge transfer from chalcogen compounds to graphene and successfully open a band gap in graphene. This modulation of the electronic structure mainly arises from symmetry breaking caused by interfacial coupling. 

In summary, this study systematically elucidates the structure–performance relationship of graphene/chalcogenide heterojunctions from microscopic to macroscopic scales, revealing their electric-field-sensitive mechanisms and laying a solid theoretical and experimental foundation for the subsequent design of high-performance electric-field-sensitive materials and devices. Finally, we acknowledge certain limitations in the current study. Theoretically, while the standard PBE functional effectively captures the trends in electronic structure, it is known to underestimate the absolute bandgap values compared to hybrid functionals. Experimentally, the observed sensitivity enhancement (~7.6%), while validating the heterojunction mechanism, is relatively modest. Future work will focus on optimizing the filler concentration and microstructure to further amplify this gain, alongside conducting comprehensive system-level stability assessments.

## Data Availability

The data presented in this study are available on request from the corresponding author.
